# Hes1 Increases the Invasion Ability of Colorectal Cancer Cells via the STAT3-MMP14 Pathway

**DOI:** 10.1371/journal.pone.0144322

**Published:** 2015-12-09

**Authors:** MT Weng, PN Tsao, HL Lin, CC Tung, MC Change, YT Chang, JM Wong, SC Wei

**Affiliations:** 1 Department of Internal Medicine, Far-Eastern Memorial Hospital, New Taipei, Taiwan; 2 Department of Chemical Engineering & Materials Science, Yuan-Ze University, Taoyuan, Taiwan; 3 Department of Internal Medicine, National Taiwan University Hospital and College of Medicine, Taipei, Taiwan; 4 Department of Pediatrics, National Taiwan University Hospital and College of Medicine, Taipei, Taiwan; 5 Department of Integrated Diagnostics & Therapeutics, National Taiwan University Hospital and College of Medicine, Taipei, Taiwan; The University of Hong Kong, CHINA

## Abstract

The Notch pathway contributes to self-renewal of tumor-initiating cell and inhibition of normal colonic epithelial cell differentiation. Deregulated expression of Notch1 and Jagged1 is observed in colorectal cancer. Hairy/enhancer of split (HES) family, the most characterized targets of Notch, involved in the development of many cancers. In this study, we explored the role of Hes1 in the tumorigenesis of colorectal cancer. Knocking down Hes1 induced CRC cell senescence and decreased the invasion ability, whereas over-expression of Hes1 increased STAT3 phosphorylation activity and up-regulated MMP14 protein level. We further explored the expression of Hes1 in human colorectal cancer and found high Hes1 mRNA expression is associated with poor prognosis in CRC patients. These findings suggest that Hes1 regulates the invasion ability through the STAT3-MMP14 pathway in CRC cells and high Hes1 expression is a predictor of poor prognosis of CRC.

## Introduction

Colorectal cancer (CRC) is the third most common cancer [1] and surgical resection is the standard treatment for early stage CRC. Chemotherapies including oxaliplatin, irinotecan, and flouracil are wildly used to treat the advanced disease [2]. Therapies against vascular endothelial growth factors or their receptors, such as bevacizumab [3], aflibercept [4], and regorafenib [5], prolong survival of patients with advanced colorectal cancers. However, despite advances in surgical resections and systemic therapies, including adjuvant chemotherapies, many patients still die of CRC, and colorectal cancer is fourth in cancer related deaths worldwide [1].

Genetic and epigenetic alterations drive the initiation and progression of the adenoma–carcinoma sequence in colorectal cancer [6] and aberrant activation of the Notch1 signaling is one of the identified pathway [7, 8]. Notch signaling contributes to the self-renewal of tumor-initiating cells, expansion of the intestinal progenitor pool, and inhibition of normal colonic epithelial cell differentiation [9–11]. Notch signaling is activated through the binding of two groups of ligands, Jagged (Jagged1, 2) and Delta-like (Dll1, 3, 4). Higher expression of Notch1 and Jagged1 mRNA and their proteins were observed in CRC tissues compared to adjacent non-tumor tissue [12]. Furthermore, Notch1 protein expression was positively correlated with tumor stage [12] and pathology grade (high expression Notch1 in poorly differentiated carcinoma) [8, 13].

One of the most characterized targets of Notch is the hairy/enhancer of split (HES) family [14]. This target shows promise in inducing intestinal tumor cell differentiation [15]. Overexpression of Hes1 has been associated with the development of pancreatic [14, 16, 17], breast [18] and ovarian [19] cancers and its down-regulation results in accelerated differentiation and decreased cellular proliferation in several cancer models [20, 21]. To date, there has been scant data about Hes1 expression in colorectal cancer [7, 22, 23]. Since high Notch expression is observed in colorectal cancer and is associated with the tumor stage, we were interested in knowing whether Hes1 is involved in the tumorigenesis of colon adenocarcinoma.

In the present study, we explored the expression of Hes1 in human colorectal cancer and correlated its expression with the clinical results. High Hes1 mRNA expression is associated with poor prognosis in CRC patients. In the functional analysis, we found that suppression of Hes1 expression induces more CRC cell senescence and decreases the invasion ability of CRC cells through regulating MMP14 expression.

## Material and Methods

### Cell cultures

Human 293 T cells and colon cancer cell lines: HCT116, Caco2 and SW48 were obtained from the American Type Culture Collection (ATCC, Manassas, VA). They were maintained in DMEM (Gibco-BRL, Gaithersburg, MD) supplemented with 10% fetal bovine serum and 1% penicillin/streptomycin 100 μg/ml streptomycin. Cells were grown at 37°C in a 5% CO2 atmosphere within a humidified incubator.

### Reagents

Plasmid EF.hHES1.Ubc.GFP and plasmid EF.deltaBHES1.Ubc.GFP were acquired from Linzhao Cheng’s lab [24] via addgene Inc. (Cambridge, MA). The Flag-Hes1 was generated by digesting EF.hHES1.Ubc.GFP with BamHI and XhoI, and inserting the digested plasmid into the multiple cloning sites of the pcDNA4-TAG vector (Invitrogen, Carlsbad, CA). Stattic was obtained from Sigma-Aldrich (St. Louis, MO). Control siRNA and Hes1 siRNA were obtained from Qiagen (Venlo, Netherlands). Lipofetamine RNAiMAX (Invitrogen) was used as an siRNA transfection reagent. The protocol was done according to the manufacturer’s instructions at a final concentration of 10nM.

### Western Blot

Western blots were performed as previously described [24]. The primary antibodies used were Hes1 (Millipore, Billerica, MA), MMP14 (Abcam, Cambridge, UK), STAT3, phosphor-STAT3 (Cell Signaling Technology, Danvers, MA), Flag and Actin (Sigma-Aldrich). Membranes were then incubated with a specific primary antibody overnight, washed, then incubated with an appropriate secondary antibody conjugated to horseradish peroxidase, and developed using ECL (PerkinElmer Life Sciences, Waltham, MA).

### RNA extraction and real-time polymerase chain reaction (RT-PCR)

Total RNA from patient samples was extracted with RNA extraction kit (Qiagen) from tissue homogenized with Trizol (Invitrogen). Total RNA from the cell lines was extracted with an RNeasy Mini kit (Qiagen). The RNA was reverse transcribed to DNA using the High Capacity cDNA Reverse Transcription kit (Applied Biosystems, Foster City, CA). Expression of Hes1 and MMP2, MMP3, MMP7, MMP9 and MMP14 mRNA were evaluated by using Power SYBR Green PCR Master Mix (Applied Biosystems). GAPDH mRNA was used as an endogenous control. Expression of RNA was analyzed using the 2-ΔΔCt method. Primers for mRNA expression were demonstrated in S1 Table.

### Proliferation assay

Cell viability was determined by the MTT [3-(4,5-dimethylthiazol-2-yl)-2,5- diphenyltetrazoliumbromide, Sigma-Aldrich Co.] assay.

### In vitro invasion assay

The invasive activity was measured by using a membrane invasion culture system in which a polycarbonate membrane with 8-μm pores (Millipore, Billerica, MA) coated with Matrigel (R&D Systems Inc., Minneapolis, MN) at 5 mg/mL was placed between the upper and lower wells of a membrane invasion culture system chamber. Following that, 5 × 10^4^ HCT116 or SW48 cells were placed into each upper well of the chamber. After a 48 hour incubation at 37°C, cells that had migrated through the coated membrane were removed from the lower chamber with 1 Mm EDTA in PBS and dot blotted onto a polycarbonate membrane with 3 μm pores. Blotted cells were stained with Giemsa (Sigma Chemical Co., St. Louis, MO), and the number of cells on each blot was counted under a microscope at a magnification of 50×. Each experiment was performed three times, and each sample was assayed in triplicate.

### SA-β-gal activity assay

Caco2 cells were treated with a Senescence β-Galactosidase Staining Kit (Cell Signaling). The protocol was done according to manufacturer’s instruction. Briefly, 2 *10^5^ Caco2 cells were transfected with siRNA and plated in a 35 mm well for 24 hrs. The cells were then washed with PBS, fixed in 1X fixative solution for 10 min at room temperature, and rinsed twice with PBS. The cells were then incubated with β-galactosidase staining solution for 10 hr at 37°C. Blue-stained cells were counted at 400× magnification.

### Human CRC tissue

Colorectal cryosections were prepared from colorectal cancer surgical samples which were collected from September 2000 to June 2003 after obtaining the written informed consent. This study was approved by the Ethics Review Board at the National Taiwan University Hospital. All tissues were freshly frozen or immersed in optimal cutting temperature compound (OCT) (Ames Company, Elkhart, IN), and kept at −80°C until use. Clinical staging of cancers was determined based on the UICC-TNM classification.

### Statistical analysis

Comparisons of variables between the two groups were performed using two tailed Student’s t test with expressed as means + standard error of mean (S.E.M). All experiments were performed at least in triplicate. Paired T-tests were used for comparing tumor and non-tumor mRNA expression. We used the Spearman method to analyze correlations between Hes1 and MMP14. High and low Hes1 and MMP14 expression were defined according to the median expression level in each group. The survival was calculated using the Kaplan–Meier method and compared using the log-rank test. P values of less than 0.05 were regarded as significant.

## Result

### Knocking down the Hes1 gene impaired invasion ability

**Fig 1:**
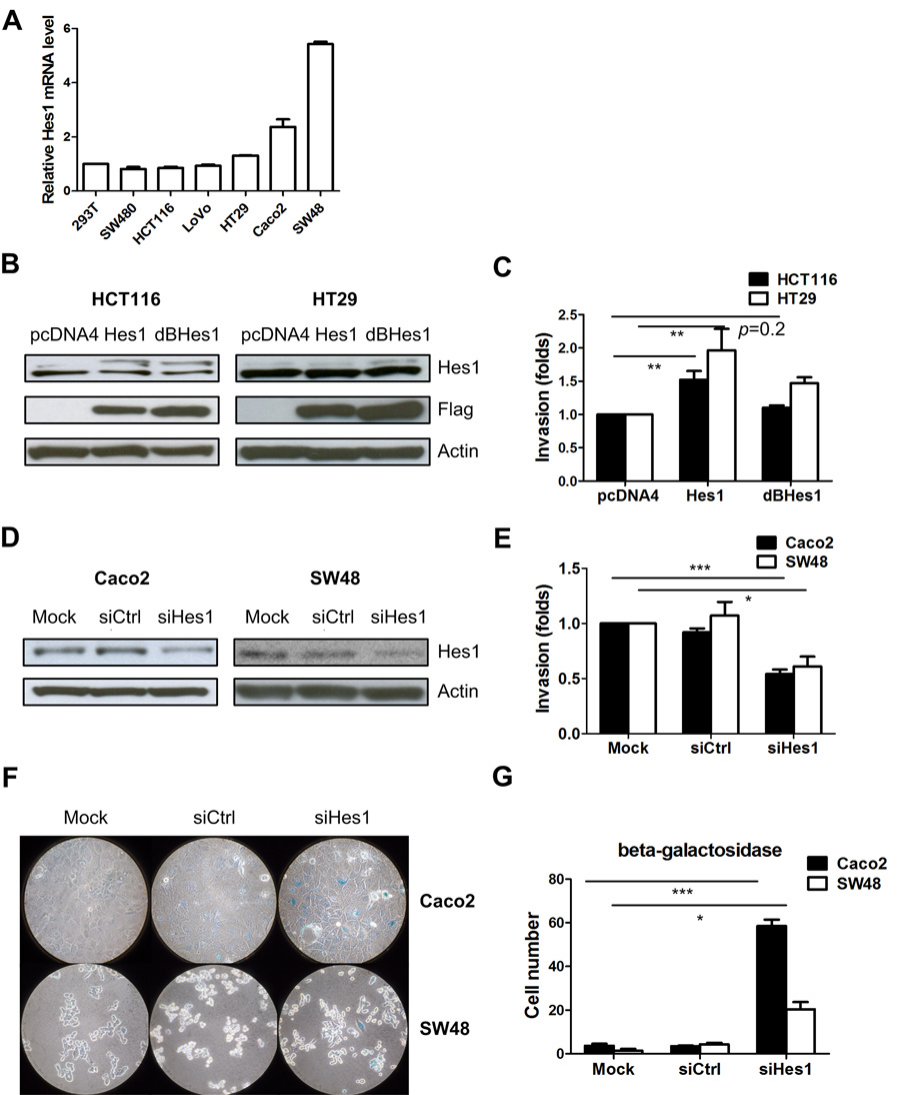
Overexpression of Hes1 increases cell invasion ability. (A) Hes1 mRNA expression in the colon cell lines. (B) Hes1 and Flag protein expression upon overexpression of Hes1 and mutant Hes1 in HCT116 and HT29 cells. (C) Cell invasion ability changes upon overexpression Hes1 and mutant Hes1 in HCT116 and HT29 cells. (D) Expression of the Hes1 protein in Caco2 and SW48 cells upon siRNA Hes1 transfection. (E) Cell invasion ability changes upon siRNA Hes1 transfection. (F) Representative images of senescence β-Galactosidase in the Caco2 and SW48 cells. (G) Quantification of senescence β-Galactosidase positive cells in Fig 1F. (* p < 0.05, ** p < 0.01, *** p < 0.005).

To explore the roles of Hes1 in colon cancer cells, we examined Hes1 expression levels in 6 colon cancer cell lines using quantitative RT-PCR and found high Hes1 expression levels in CaCo2 and SW48 cells and low Hes1 expression levels in HCT116 cells (Fig 1A). We then stably expressed HES1 or a mutant Hes1 lacking the DNA-binding domain, in HCT116 and HT29 colon cancer cells (Fig 1B). We observed higher invasion ability in the Hes1 overexpressing cells compared to cells infected with the control pcDNA4 plasmid. We did not observe increased invasion ability in HCT116 and HT29 cells expressing mutant Hes1, suggesting that the DNA binding domain is important for the invasion ability of colon cancer cells (Fig 1C). We next depleted Hes1 in the SW48 and Caco2 cells using siRNA (Fig 1D), and observed less invasion in cells transfected with Hes1 siRNA than those with control siRNA (Fig 1E). To further determine the role of Hes1 in cancer characteristic of CRC cells, a proliferation assay was performed and it showed no significant change after Hes1 depletion (S1 Fig).

### Depleting the Hes1 gene induced more CRC cells into senescence

It has been reported that Hes1 is required for the reversibility of cellular quiescence and it prevents premature senescence in quiescent fibroblasts [25]. We knocked down Hes1 in SW48 and Caco2 cells and observed significantly more beta-galactosidase expression in Hes1 knocked down cells, suggesting more senescence in the Hes1depleted cells (Fig 1F and 1G).

### Knockdown of the Hes1 gene decreases invasion through regulating MMP14

**Fig 2:**
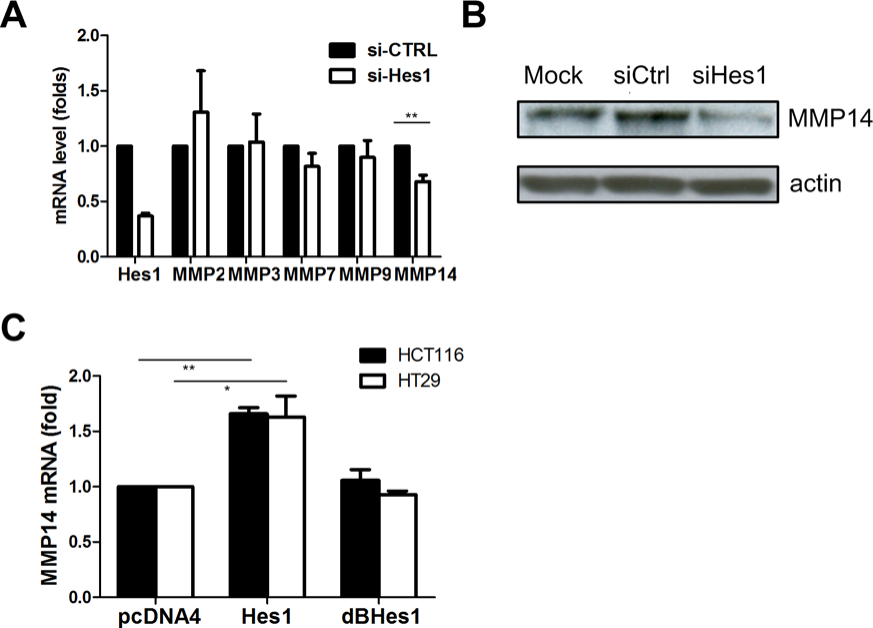
Knockdown of Hes1 reduces MMP14 mRNA expression. (A) A series of MMP mRNA expression upon siRNA Hes1 transfection in Caco2 cells. (B) MMP14 protein decrease upon siRNA Hes1 transfection in SW48 cells (C) MMP14 mRNA expression upon Hes1 and mutant Hes1 overexpression in the HCT116 and HT29 cells. (* p < 0.05, ** p < 0.01)

As several matrix metalloproteinases (MMPs) mediate invasion of cancer cells [26], we studied the expression of a panel of MMPs in colon cancer cells upon Hes1 knockdown. We observed that only MMP14 expression level decreased upon Hes1 knockdown at the mRNA level in Caco2 cells (Fig 2A). We then found knock-down Hes1 lead to MMP14 protein level decrease in SW48 cells (Fig 2B). Conversely, over expression of Hes1 led to an increase of MMP14 mRNA expression (Fig 2C). We ectopically expressed a mutant HES1 gene, which carries a truncation at its function domain, and we did not observe the increase in MMP14 in the HCT116 and HT29 cells (Fig 2C).

### Hes1 regulated MMP14 expression depends on the STAT3 pathway

**Fig 3:**
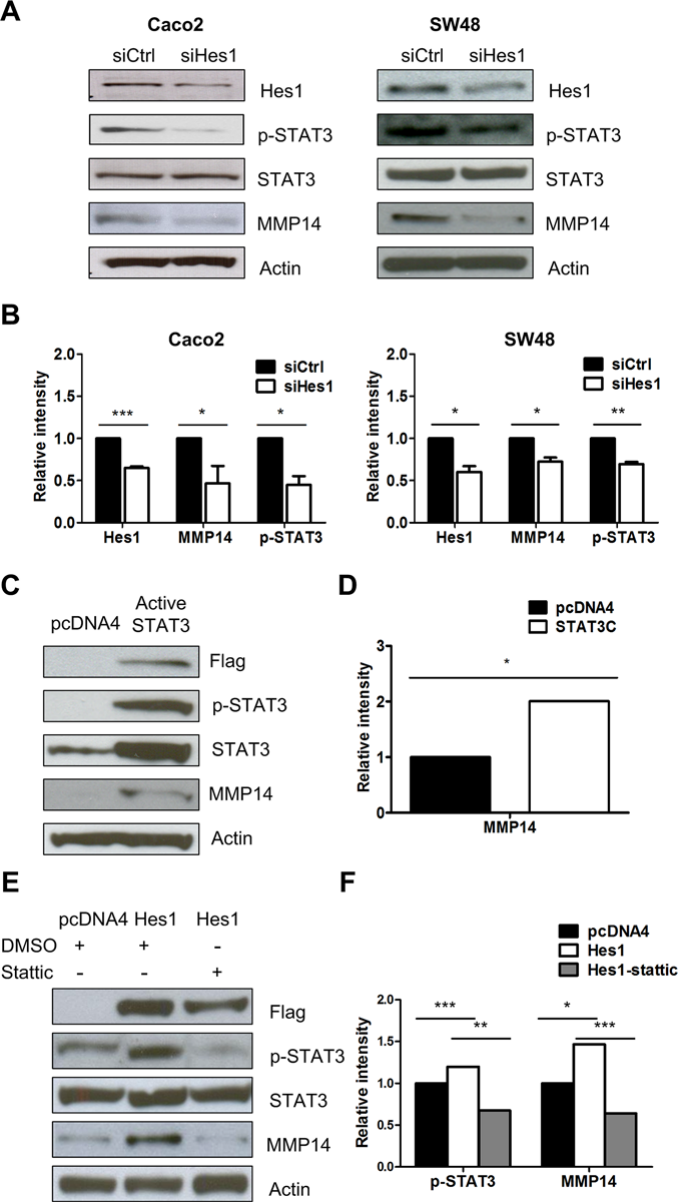
Knockdown of Hes1 decreased MMP14 expression through the STAT3 pathway (A). Expression of phorphorylated STAT3, total STAT3 and MMP14 protein upon siRNA Hes1 transfection in Caco2 and SW48 cells. (B) Quantification of Fig 3A. (C) MMP14 protein expression upon overexpression of constitutively active STAT3 in the HCT116 cells. (D) Quantification of Fig 3C. (E) phorphorylated STAT3 and MMP14 protein expression increased after Hes1 overexpression and was reduced after treating with stattic 15 uM for 24 hours in HCT116 cells. (F) Quantification of Fig 3E (* *p*< 0.05; ** p < 0.01; *** p < 0.005).

It was reported that Hes1 activates signal transducers and activators of the transcription 3 (STAT3) pathway [27, 28], and that STAT3 controls MMP1 expression in colon cancer cells [29, 30]. We therefore evaluated whether STAT3 activity plays a role in HES1’s regulation of MMP14 in colon cancer cells. Hes1 depletion by siRNA knockdown reduced MMP14 protein expression in the Caco2 and SW48 cells. Hes1 depletion resulted in a decrease of STAT3 phosphorylation and no change of total STAT3 in the Caco2 and SW48 cells (Fig 3A and 3B). Ectopically overexpressing a FLAG tagged constitutively active STAT3 plasmid, resulted in increased MMP14 protein levels in HCT116 cells (Fig 3C and 3D). Ectopic overexpression of Hes1 increased MMP14 expression as well as STAT3 phosphyorylation in the HCT116 cells. Furthermore, STAT3 phosphorylation and MMP14 protein expression was reduced when cells were treated with stattic, a STAT3 activation inhibitor (Fig 3E and 3F). Our findings suggest that HES1 regulate MMP14 expression through up-regulating STAT3 activity in colon cancer cells.

### Hes1 and MMP14 expression are unrelated to tumor stage

**Fig 4:**
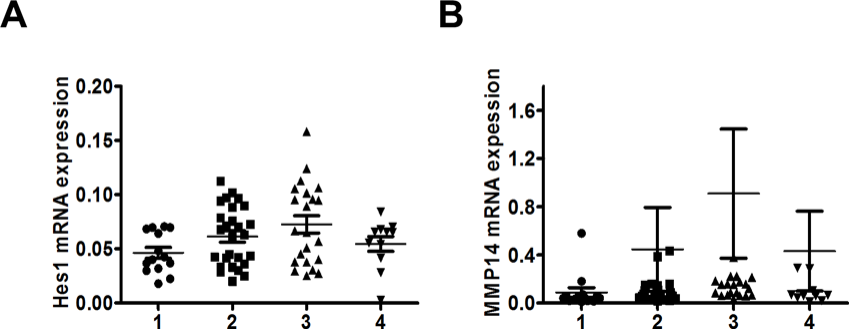
Hes1 and MMP14 mRNA expression was unrelated to tumor stage. (A) Hes1 mRNA (*p* = 0.06 by One way ANOVA) and (B) MMP14 mRNA expression (*p* = 0.60 by One way ANOVA) was not correlated with tumor stage.

As Notch1 expression is associated with tumor stage, we evaluated the expression of Hes1 in human CRC tissues. We analyzed 74 paired human colon adenocarcinoma and their adjacent non-tumor colon tissues from the National Taiwan University Hospital patient cohort (Table 1). We found that the expression level of Hes1 (Fig 4A) or MMP14 mRNA (Fig 4B) was unrelated to the tumor stage.

**Table 1:** Clinical features of colorectal patients.

Variable	No. of patients	%
**CRC patients**	74	
**Age (years)**		
Median	72.5	
Range	26–94	
**Sex**		
Male	39	53%
Female	35	47%
**Stage**		
I	14	19%
II	27	36%
III	22	30%
IV	11	15%

### High expression of Hes1 in CRC is associated with shorter survival

**Fig 5:**
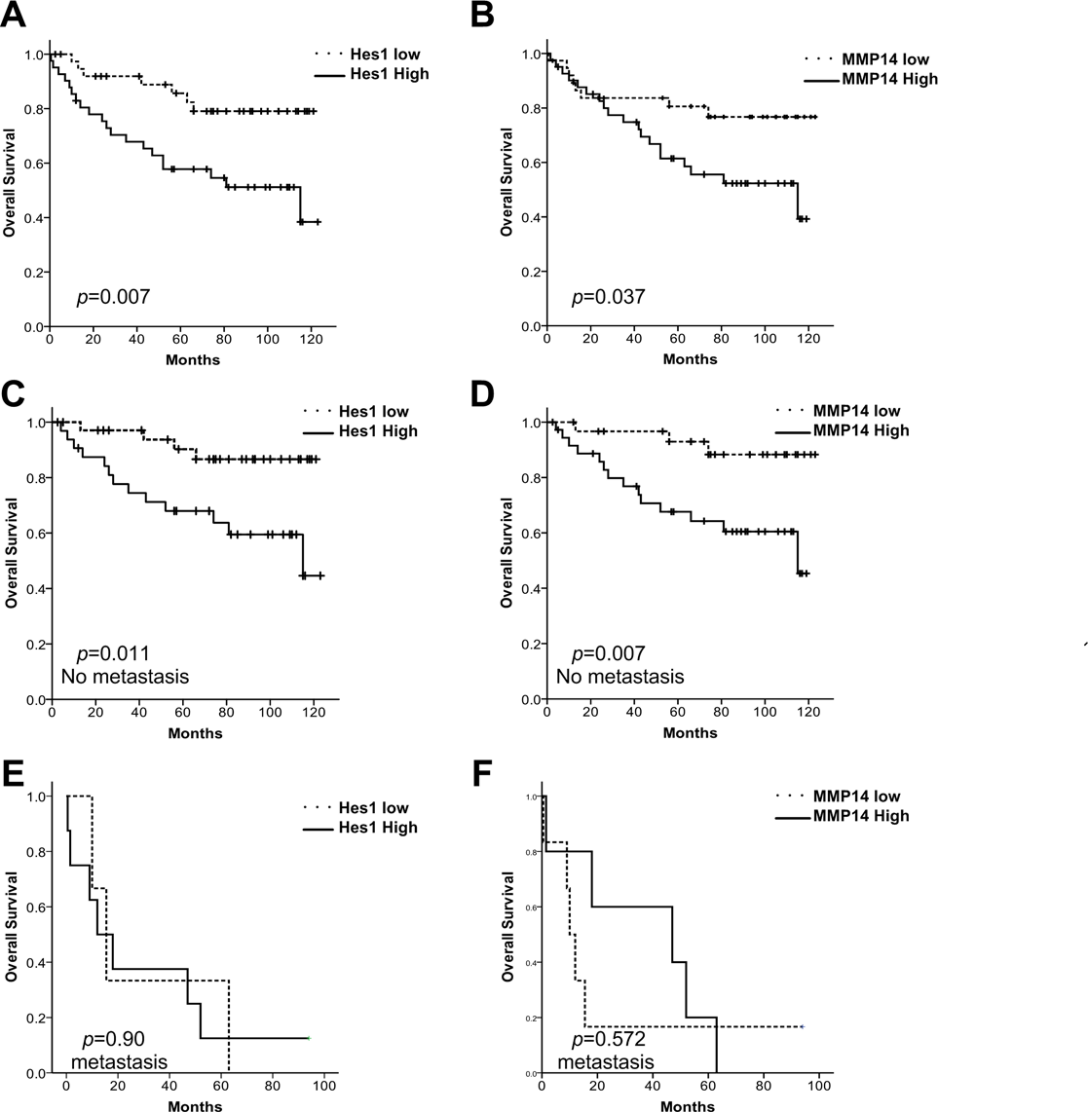
Clinical associations between Hes1 and MMP14 expression and survival in CRC patients. Patients were divided into Hes1/MMP14 low and high groups based on median ERH/MMP14 expression and their survival was plotting using the Kaplan–Meier analysis (uptick indicates censoring events). (A and B) CRC patient with high Hes1 and MMP14 expression is associated with poor survival. (C and D) In the subset of patients with stage I-III CRC, high Hes1 and MMP14 expression is significantly associated with poor survival. (E and F) In the subset of patients with stage IV CRC, Hes1 and MMP14 expression is not associated with survival.

We further investigated whether Hes1 and MMP14 expressions correlate with patient survival. Hes1 mRNA expression and MMP14 mRNA expression were positively correlated in tumor tissue (correlation coefficiency = 0.238, p = 0.035). High Hes1 expression in the tumors was correlated with shorter survival times (Fig 5A). High MMP14 was also correlated with a shorter survival (Fig 5B).

We further stratified these patients according to their metastatic diseases. For patients with early or locally advanced diseases, high Hes1 or high MMP14 expression was associated with significantly shorter survival. However, Hes1 and MMP14 expression levels were unrelated to survival for patients with metastatic diseases (Fig 5C–5F). As MMP plays a role in cancer invasion and metastasis, it may be less prognostic for patients with tumors that were already metastasized on diagnosis.

According to the multivariate Cox regression analyses, Hes1 (HR, 2.41; 95% CI, 1.07 to 5.43) and MMP14 (HR, 2.36; 95% CI, 1.02 to 5.46) remained positively associated with deaths from colorectal cancer. Metastasis was also significantly associated (HR, 10.05; 95% CI, 4.11 to 24.60) with deaths from colorectal cancer (Table 2). Regarding the interaction between Hes1 and metastasis, patients presented high Hes1 still had an increased risk of death from colorectal cancer.

**Table 2:** Multivariate analysis of selected factors for deaths from colorectal cancer.

Variable	Hazard ratio	*p*-value	95% CI
**HES1**			
Metastasis	10.05	<0.001	4.11–24.60
High Hes1	2.41	0.034	1.07–5.43
Age	1.01	0.38	0.98–1.05
sex	1.24	0.61	0.55–2.81
**MMP14**			
Metastasis	8.36	< 0.001	3.54–19.75
High MMP14	2.36	0.046	1.02–5.46
Age	1.02	0.18	0.99–1.06
sex	1.44	0.39	0.63–3.27

## Discussion

The homeostasis of self-renewal, proliferation and differentiation of stem and progenitor cells are regulated by Notch, WNT, FGF and the Hedgehog signaling pathways [31–33]. In the intestine, these pathways are also involved in villi formation and colorectal tumor development [9, 34, 35]. The WNT pathway exhibits cross-regulatory interactions with the Notch pathway [36, 37]. APC^Min^ (multiple intestinal neoplasia) mice, in which the APC gene carries a truncation mutation at codon 850 and the WNT pathway is dysregulated, can develop hundreds of small intestine and colonic polyps [38]. Notch activation is essential for colonic adenoma formation in APC^Min^ mice [10]. Inhibition of Hes1 in APC^Min^ mice has decreased tumor cell proliferation and has led to tumor cell differentiation into intestinal epithelial cells [15].

Hes1 plays an important role in CRC carcinogenesis. It binds directly to the promoter of the HATH1 gene and suppresses Hath1 expression [39]. Hath1 is a tumor suppressor which inhibits proliferation and anchorage-independent ability of colon cancer cells [40, 41] and promotes intestinal secretory cell differentiation [42]. It is well known that Hes1 prevents premature senescence in quiescent fibroblasts [25]. Here, we demonstrated that depleting Hes1 in colon cancer cells didn’t affect the cell proliferation but increased beta-galactosidase expression, indicating that Hes1 depletion induces colon cancer cell senescence. Down-regulation of Hes1 induced senescence has also been reported in hepatocellular cells through regulating CDKN1C/p57 [43].

Next, we characterized that Hes1 promoted cell invasion by increasing MMP14 expression. MMP14 is a key protease in cell migration/invasion and angiogenesis [44, 45]. High expression of MMP14 was reported in lung cancer and glioma [46, 47]. Hes1 interacts with STAT3 and promotes its phosphorylation [28]. Furthermore, STAT3 were reported to up-regulate MMP-2 and MMP-9 expression and activities via directly binding to their promoter [48–50]. In this study, we also observed that STAT3 overexpression increased expression of MMP14 (Fig 3). Our findings suggest that up-regulation of MMP14 by Hes1 in CRC cells is dependent on the STAT3 pathway.

Finally, we observed that both high Hes1 and MMP14 expression were correlated with poor survival. Similar result was found in the subgroup of patients with early or locally advanced CRC, but not in patients that the tumor has metastasized. In Veenendaal’s study, they observed higher Hes1 expression in primary colon cancers but lost expression in regional and distant metastases [23]. This finding suggested reduced Notch activity in secondary colorectal tumors and may explain why the Hes1 expression level is not associated with survival when tumor has metastasied in our study. In contrast, Reedijk et al. previously reported that high Hes1 is unrelated to survival of CRC patients by analyzing 130 microarray data from US National Cancer Institute Colon Cancer Family Registry. High expression was defined as the top quartile of the Allred scores [7]. Although Reedijk’s result conflicts with our finding, the tumor stage, high expression definition and methodological approach are different in these two studies. The real prognostic role of Hes1 in CRC warrants further confirmation.

In conclusion, aberrant activation of the Notch pathway inhibits cellular differentiation and contributes to carcinogenesis. Hes1 is a key transcriptional repressor and essential regulators for the Notch pathway. Here, we demonstrated that overexpression of Hes1 increases cell invasion ability through the STAT3-MMP14 pathway. Hes1 expression is correlated with MMP14 expression in CRC and is a predictor for patient survival. Targeting the Notch-Hes1 pathway could be exploited for therapeutic purposes in Notch activated cancers.

## Supporting Information

S1 Fig (A) Cell viability change upon siRNA Hes1 transfection in the SW48 cells. (TIF)

S1 Table Primers for RT- PCR. (DOCX)
