# Supplementary material for: Hes1 Increases the Invasion Ability of Colorectal Cancer Cells via the STAT3-MMP14 Pathway
Source: PLoS One. 2015 Dec 9;10(12):e0144322. doi: 10.1371/journal.pone.0144322 (PMC4674118; doi:10.1371/journal.pone.0144322)
Supplement: S1 Table — (DOCX) [file pone.0144322.s002.docx]

**S1 table. Primers for RT- PCR .**

|  | Forward sequence | Reverse sequence |
| --- | --- | --- |
| Hes1 | TCAACACGACACCGGATAAAC | GCCGCGAGCTATCTTTCTTCA |
| MMP2 | GATACCCCTTTGACGGTAAGGA | CCTTCTCCCAAGGTCCATAGC |
| MMP3 | AGTCTTCCAATCCTACTGTTGCT | TCCCCGTCACCTCCAATCC |
| MMP7 | GAGTGAGCTACAGTGGGAACA | CTATGACGCGGGAGTTTAACAT |
| MMP9 | GGGSCGCAGACATCGTCATC | TCGTCATCGTCGAAATGGGC |
| MMP14 | CGAGGTGCCCTATGCCTAC | CTCGGCAGAGTCAAAGTGG |
